# Patients with Rheumatoid Arthritis Show Altered Lipoprotein Profiles with Dysfunctional High-Density Lipoproteins that Can Exacerbate Inflammatory and Atherogenic Process

**DOI:** 10.1371/journal.pone.0164564

**Published:** 2016-10-13

**Authors:** Jae-Yong Kim, Eun-Young Lee, Jin Kyun Park, Yeong Wook Song, Jae-Ryong Kim, Kyung-Hyun Cho

**Affiliations:** 1 Dept of Medical Biotechnology, Yeungnam University, Gyeongsan, 712–749, Republic of Korea; 2 Research Institute of Protein Sensor, Yeungnam University, Gyeongsan, 712–749, Republic of Korea; 3 BK21plus Program Serum Biomedical Research and Education Team, Yeungnam University, Gyeongsan, 712–749, Republic of Korea; 4 BK21plus Program, Department of Molecular Medicine and Biopharmaceutical Sciences, Graduate School of Convergence Science and Technology, and College of Medicine, Medical Research Center, Seoul National University, Seoul, Republic of Korea; 5 Division of Rheumatology, Department of Internal Medicine, Seoul National University Hospital, Seoul, Republic of Korea; 6 Department of Biochemistry and Molecular Biology, College of Medicine, Yeungnam University, Daegu,705–717, Republic of Korea; Medical University Innsbruck, AUSTRIA

## Abstract

**Objective:**

In order to identify putative biomarkers in lipoprotein, we compared lipid and lipoprotein properties between rheumatoid arthritis (RA) patients and control with similar age.

**Methods:**

We analyzed four classes of lipoproteins (VLDL, LDL, HDL_2_, HDL_3_) from both male (n = 8, 69±4 year-old) and female (n = 25, 53±7 year-old) rheumatoid arthritis (RA) patients as well as controls with similar age (n = 13).

**Results:**

Although RA group showed normal levels of total cholesterol (TC), low-density lipoprotein (LDL)-cholesterol, and glucose, however, the RA group showed significantly reduced high-density lipoprotein (HDL)-C level and ratio of HDL-C/TC. The RA group showed significantly elevated levels of blood triglyceride (TG), uric acid, and cholesteryl ester transfer protein (CETP) activity. The RA group also showed elevated levels of advanced glycated end (AGE) products in all lipoproteins and severe aggregation of apoA-I in HDL. As CETP activity and TG contents were 2-fold increased in HDL from RA group, paraoxonase activity was reduced upto 20%. Electron microscopy revealed that RA group showed much less HDL_2_ particle number than control. LDL from the RA group was severely oxidized and glycated with greater fragmentation of apo-B, especially in female group, it was more atherogenic via phagocytosis.

**Conclusion:**

Lipoproteins from the RA patients showed severely altered structure with impaired functionality, which is very similar to that observed in coronary heart patients. These dysfunctional properties in lipoproteins from the RA patients might be associated with high incidence of cardiovascular events in RA patients.

## Introduction

Rheumatoid arthritis (RA) and systemic lupus erythematosus (SLE) are long lasting autoimmune diseases. RA is a chronic inflammatory disorder of the synovium in joints, causing bone destruction with progressive pain. RA may result in low red blood cell counts, inflammation around the lungs, and inflammation around the heart [1]. Patients with RA are at significantly increased risk of cardiovascular disease (CVD) [2] and stroke [3], including myocardial infarction and sudden cardiac death, which is not explained by traditional CVD risk factors such as cholesterol and low-density lipoproteins (LDL).

Although oxidized LDL (oxLDL) is a traditional biomarker of atherosclerosis, many reports have suggested that patients with RA and diabetes mellitus also showed high level of oxLDL [4]. RA patients showed elevation of advanced glycation end (AGE) products in immunoglobulin G (IgG) [5] and diabetic patients showed glycation in hemoglobin and albumin. It has been suggested previously that elevation of AGE in lipoproteins is independent biomarkers in aging and aging related disease such as CVD [6] and diabetes [7]. Our group reported that the elderly group showed an increase in AGE in high-density lipoprotein (HDL) with truncation of apolipoprotein A-I (apoA-I), resulting in production of dysfunctional and pro-inflammatory HDL [8, 9].

It has been well established that pro-inflammatory HDL, which is associated with glycation and oxidation, is a biomarker of atherosclerosis in patients with RA [10]. Regarding lipid parameters, RA patients showed increased serum TG levels and decreased HDL-C levels, whereas serum TC levels are not different [11]. Regarding protein level, apoA-I was reduced by 12% while apo-B increased by 14% in RA patients [11]. Especially, myeloperoxidase (MPO) activity increased up to 2-fold as cholesterol efflux decreased [12].

In addition to HDL-C quantity, the quality of HDL has recently been shown to prevent CVD and diabetes [13]. Although RA is associated with dysfunctional and pro-inflammatory HDL, there has been no report elucidating lipoprotein properties and cardiovascular risk in RA patients. To identify unique lipoprotein properties associated with RA in blood, we analyzed lipoprotein compositions and functions. This study compared lipid and lipoprotein properties between RA and control in male and female group. We compared four classes of lipoproteins, very low-density lipoprotein (VLDL), LDL, HDL_2_, and HDL_3_, which were individually separated from subjects, based on their structural and functional modifications to compare biomarkers of lipoprotein parameters.

## Materials and Methods

### Patients and ethics statement

We recruited male and female patients with RA who visited Seoul National University Hospital. Patients, who were diagnosed with RA according to the 1987 American College of Rheumatology classification criteria [14] were enrolled in this study. Average duration of RA was 10±2 and 15±4 years for male and female patients respectively. Around 50% of patients were taken corticosteroid with 5.0±3.1 and 2.2±2.7 mg/day for male (n = 4 in 8) and female (n = 13 in 25) patients, respectively. Heavy alcohol consumers (>30 g EtOH/day) and those who had consumed any prescribed drugs to treat hyperlipidemia, diabetes mellitus, or hypertension were excluded. All subjects had unremarkable medical records without illicit drug use or past histories of systemic diseases. All patients were informed about the study and signed the informed consent prior to enrollment in the study, and the Institutional Review Board at the Medical Center of Seoul National University (Seoul, South Korea) approved the protocol. The recruitment study period was from January 2013 to November 2015.

RA patients were classified according to their disease activity scores using a 28 joint count (DAS-28) [15]. Moderate-high disease activity was defined as a DAS-28 score >3.2 (Active RA, n = 13), while low disease activity was defined as a DAS-28 score <3.2 (Inactive RA, n = 20). Beside corticosteroids, major medications for RA patients were Methotrexate (MTX, n = 28) and NSAIDs (n = 25). Minor medications were sulfasalazine (n = 8), hydroxychloroquine (n = 9), and leflonomide (n = 5). Only two patients took a statin while one patient took an ACE inhibitor. Control subjects were voluntarily recruited from those who attended the Health Promotion Program of Seoul National University Hospital for regular health examinations. All were interviewed, and absence of clinical evidence of RA was confirmed by thorough examination of medical history and physical examination

### Blood analysis

Blood was obtained from both RA patients and control after overnight fasting state immediately before the procedure. Blood was collected using a vacutainer (BD sciences, Franklin Lakes, NJ) containing EDTA (final 1 mM). Plasma was isolated by low speed centrifugation and stored at -80°C until analysis. Blood parameters such as lipids and glucose concentrations were determined using an automatic blood analyzer (Chemistry analyzer AU4500 Olympus, Tokyo, Japan) and by manual determination using a commercially available assay kit. Plasma total cholesterol (TC), HDL-C, triglycerides (TG), and glucose levels were determined using commercial assay kits (Wako Pure Chemical, Osaka, Japan). LDL-cholesterol (LDL-C) was calculated using Friedewald formula, LDL-C = TC-(HDL-C +(TG/5)). Blood uric acid level was analyzed according to the previous method by Caraway [16].

### Isolation of lipoproteins

Very low-density lipoproteins (VLDL, d<1.019 g/mL), LDL (1.019<d<1.063), HDL_2_ (1.063<d<1.125), and HDL_3_ (1.125<d<1.225) were isolated from individual patients and control plasma via sequential ultracentrifugation [17], with density adjusted by addition of NaCl and NaBr in accordance with standard protocols. Samples were centrifuged for 24 hours at 10°C at 100,000g using a Himac CP-90α (Hitachi, Tokyo, Japan) at the Instrumental Analysis Center of Yeungnam University.

To analyze individual lipoproteins, total cholesterol (TC) and triglyceride (TG) levels were measured using commercially available kits (cholesterol, T-CHO and TG, Cleantech TS-S; Wako Pure Chemical, Osaka, Japan). Protein concentrations in lipoproteins were determined via Lowry protein assay, as modified by Markwell *et al* [18]. To assess the degree of oxidation of individual LDL, concentration of oxidized species in LDL was determined by the thiobarbituric acid reactive substances (TBARS) method using malondialdehyde as a standard [19].

To compare the extent of glycation between groups, content of AGEs in individual lipoproteins was determined from fluorescence measurements at 370 nm (excitation) and 440 nm (emission), as described previously [20].

### Copper-mediated LDL-oxidation

To assess copper-mediated LDL oxidation, 300 μg of LDL was incubated with 5 μM CuSO_4_ for up to 3 hours. During incubation, the quantity of formed conjugated dienes was monitored by measuring the absorbance at 234 nm (Abs_234_) and 37°C [21] using a Beckman DU 800 spectrophotometer equipped with a MultiTemp III thermocirculator.

To verify spectroscopic data, oxLDL samples were subjected to electrophoresis on a 0.5% agarose gel to compare relative electromobilities [22]. The post-oxidative electrophoretic mobility of LDL was compared via electrophoresis on a 0.5% agarose gel due to modification of amino acids in apo-B and phospholipids by oxidation.

### Ferric reducing ability of plasma assay

The ferric reducing ability of plasma (FRAP) was determined using the method described by Benzie and Strain [23] with a slight modification, as described recently by our research group [24]. The antioxidant activities of individual HDL fractions (20 μg each in PBS) were then estimated by measuring the increase in absorbance induced by generated ferrous ions.

### Cholesteryl ester transfer assay

A rHDL-containing apoA-I and cholesteryl oleate was synthesized in accordance with the method described by Matz and Jonas [25], modified by Cho [26] using trace amounts of [^3^H]-cholesteryl oleate (TRK886, 3.5 μCi/mg of apoA-I; GE Healthcare). The rHDL was immobilized using CNBr-activated Sepharose 4B resin (Amersham Biosciences) for easy separation of rHDL after the reaction, in accordance with the manufacturer’s instructions. CE transfer reaction was performed in 300-μL reaction mixtures containing human plasma (20 μL) or HDL_3_ (20 μL, 2 mg/mL) as a cholesteryl ester transfer protein (CETP), [^3^H]-rHDL-agarose (20 μL, 0.25 mg/mL) as a CE-donor, and human LDL (20 μL, 0.25 mg/mL) as a CE-acceptor source, as described previously [27]. After incubation at 37°C with shaking around 200 rpm, the reaction was halted via brief centrifugation (10,000g) for 3 min at 4°C to sediment [^3^H]-rHDL-agarose. Supernatants containing CE-acceptor (150 μL) and the agarose pellet (50 μL) were then separately subjected to scintillation counting, and percentage transfer of [^3^H]-CE from [^3^H]-rHDL to LDL was calculated. For non-specific reactions, a parallel experiment was simultaneously carried out in the control tube, which includes all constituents except the CETP source.

### Paraoxonase assay

Paraoxonase-1 (PON-1) activity was determined by measuring the initial velocity of *p*-nitrophenol production at 37°C, as determined by measuring the absorbance at 405 nm (microplate reader, Bio-Rad model 680; Bio-Rad, Hercules, CA, USA), as described previously [28] with slight modification [29]. Prior to the measurement, HDL was extensively dialyzed against PBS to remove EDTA.

### ELISA and Western blot

In order to quantify plasma CETP, each wells of a polystyrene microplate (#3590; Corning Inc, Corning, NY, USA) were coated with the anti-human CETP rabbit antibody (ab19012; Abcam, Cambridge, UK) at a concentration of 0.25 μg/mL and incubated overnight at 4°C. Equally diluted each plasma sample was incubated for 2 h at room temperature. After extensive washing, anti-human CETP mouse antibody (ab2726; abcam, 1 μg/mL) was treated and incubated for 2 h at room temperature. To develop color reaction, anti-mouse IgG antibody (ab6728; abcam, 0.5 μg/mL conjugated with Horse Radish Peroxidase, was treated. For color development, 3,3',5,5' tetramethylbenzidine (TMB) substrate solution (Cat. No. 555214; BD Biosciences, Flanklin Lakes, NJ, USA) was treated and quantified by Victor X4 microplate reader (Perkin Elmer, Waltham, MA).

Apolipoprotein/lipoprotein compositions were compared via sodium dodecyl sulfate-polyacrylamide gel electrophoresis (SDS-PAGE) with identical protein loading quantities (3 μg of total protein per lane) from individual HDL_3_, and expression levels of apolipoprotein were analyzed via immunodetection. Anti-human apoA-I antibody (ab7613), anti-paraoxonase antibody (ab24261), anti-IL-6 antibody (ab6672) were purchased from Abcam (Cambridge, UK). Phosphorylated IkB antibody (Santa Cruz Biotech, Santa Cruz, CA) and beta-actin antibody (Bethyl lab A300-491A; Montgomery, TX). Relative band intensity (BI) was compared via band scanning with Chemi-Doc^®^ XRS+ (Bio-Rad, Hercules, CA) using Image Lab software (Version 5.2). Relative band intensity (BI) was compared via band scanning with Gel Doc^®^ XR (Bio-Rad) using Quantity One software (version 4.5.2).

### Electron microscopy

Transmitted electron microscopy (TEM) was performed with a Hitachi electron microscope (model H-7600; Ibaraki, Japan) operated at 80 kV, as in our previous report [30]. HDL_2_ was negatively stained with 1% sodium phosphotungastate (pH 7.4) with a final apolipoprotein concentration of 0.3 mg/mL in TBS.

### Phagocytosis of LDL into macrophages

THP-1 cells, a human monocytic cell line, were maintained and differentiated into macrophages as described previously [31]. The differentiated and adherent macrophages were then rinsed with warm PBS and incubated with 400 μL of fresh RPMI-1640 medium containing 1% FBS and 50 μL each of LDL (1 mg of protein/mL in PBS) from each group for 48 hours at 37°C in a humidified incubator. After incubation, cells were fixed and stained with oil-red O (0.67%) to visualize LDL. Cell lysate was collected and immunodetected with phospho-IκB antibody and beta-actin antibody.

### Data analysis

All data are expressed as the mean±SD from at least three independent experiments with duplicate samples. Data comparisons were assessed by Student’s *t*-test between the groups, χ^2^ test for categorical variables, and non-parametric test for comparison of means using the SPSS program (version 14.0; SPSS, Inc., Chicago, IL, USA).

## Results

### Blood profiles of RA patients

As shown in Table 1, both male and female RA patients showed normal levels of blood cholesterol (180–190 mg/dL) and glucose (94–104 mg/dL). However, plasma TG levels were 2-fold higher in RA groups, especially in females. Plasma HDL-C levels were remarkably in lower in both males (22±5 mg/dL) and females (25±6 mg/dL). RA male (RAM) subjects showed a 50% lower percentage of HDL-C/TC (12±3%) than the control (26±7%), whereas female RA subjects showed 40% lower % HDL-C than the control (23±2%). Interestingly, both males and females showed remarkably elevated plasma CETP activities around 41%, whereas the control male (CM) group showed around 23% CETP activity. CETP mass was also increased in both RA group especially female RA group showed 1.6-fold higher than control.

**Table 1:** Serum profile of rheumatoid arthritis (RA).

	CM	RAM	*p* value	CF	RAF	*p* value
	(n = 5)	(n = 8)	(vs control)	(n = 8)	(n = 25)	(vs control)
Age (years)	69±4	69±4	NS	54±1	53±7	
TC (mg/dL)	218±28	184±17	NS	210±14	191±20	NS
TG (mg/dL)	114±54	101±28	NS	91±14	181±29*	0.0287
HDL-cholesterol (mg/dL)	57±18	22±5*	0.0482	47±4	25±6*	0.0240
% HDL-cholesterol	26±7	12±3*	0.0194	23±2	14±4*	0.0283
TG/HDL-cholesterol	2.0±0.4	4.6±0.4*	0.0111	1.9±0.1	7.3±0.5**	0.0025
calculated LDL-cholesterol (mg/dL)	169±33	142±34	NS	145±12	129±19	NS
% LDL-cholesterol	77±5	77±7	NS	69±2	67±4	NS
AST (IU/L)	24±7	26±3	NS	14±0	25±6*	0.0338
ALT (IU/L)	20±8	19±4	NS	16±0	26±13	NS
Glucose (mg/dL)	105±23	104±24	NS	76±9	95±4*	0.0476
Uric acid (mg/dL)	6.0±2.4	9.0±2.3*	0.0471	4.5±2.4	8.1±3.1*	0.0152
CETP activity (% CE-transfer)	23±2	41±2***	0.0007	36±1	41±5	NS
CETP mass (ng/mL)	1.33±0.36	1.92±0.56*	0.042	1.19±0.54	1.94±0.49**	0.0016

CM, control male; RAM, rheumatoid arthritis male; CF, control female; RAF, rheumatoid arthritis female; TC, total cholesterol; TG, triglyceride; HDL, high-density lipoprotein; LDL, low-density lipoprotein; AST, aspartate aminotransferase; ALT, alanine aminotransferase; CETP, cholesteryl ester transfer protein. NS, not significant.

*, *p*<0.05;

**, *p*<0.01

**Fig 1:**
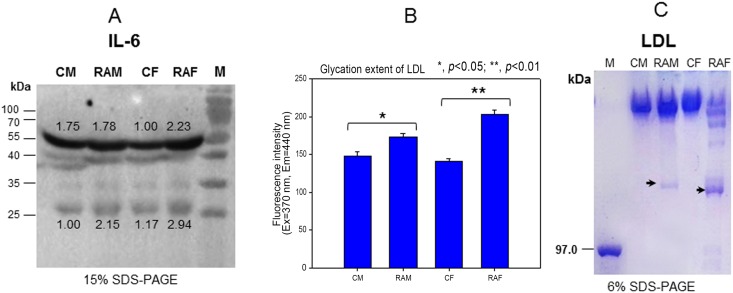
Expressional level of IL-6 in equally diluted serum (20 μg/lane) as visualized by immunodetection (A) Lower numbers indicate band intensity from Chemi-Doc analysis. Glycation extent of LDL based on fluorescence determination (B) and electrophoretic profiles of LDL as visualized by Coomassie Brilliant Blue staining (C). CM, control male; RAM, rheumatoid arthritis male; CF, control female; RAF, rheumatoid arthritis female.

More interestingly, TG/HDL-C was remarkably elevated in both RA groups; male RA and female RA groups showed 2.3- and 3.8-fold higher levels than the control, respectively. However, amount and % of LDL-C were not different between the RA and control groups for both genders. These results suggest that plasma TG and HDL-C levels were specific parameters in the RA group, whereas LDL-C was not. In both male and female RA groups, plasma AST and ALT activities were significantly elevated compared to the control. Both male and female RA groups showed 1.5- and 1.8-fold increased plasma uric acid levels compared to the control, respectively. Immunodetection revealed that plasma IL-6 level (monomer) was 2-fold more elevated in RA group than each control. However, RAF group showed 2.2-fold higher level of dimeric IL-6 than control (CF) as shown in Fig 1A.

### Lipoprotein profiles in RA patients

In VLDL, as shown in S1 Table, both male and female RA patients showed significant increases in TG contents (% wt in total amount) of 1.2 and 1.3-fold, respectively, than the control. In the RA group, TG content was 2-fold higher in the LDL fraction, whereas TC content in LDL was similar compared to the control. However, total protein (TP) in LDL was significantly reduced in the both male and female RA group. In the HDL_2_ fraction, TC content was not different between the RA and control groups in both male and female. However, TG content of HDL_2_ was 3- and 2-fold higher in the RAM and RAF groups, respectively, than the control showing a decrease in total proteins. In the HDL_3_ fraction, TC content was reduced by 27% and 57% in the RAM and RAF groups, respectively, compared with each control. However, TG content in HDL_3_ remarkably increased in the RAM and RAF groups up to 4 and 3-fold, respectively, compared to the each control. These results suggest that enrichment of TG was associated with impairment of beneficial functions in lipoproteins from the RA group to result more oxidation and proteolytic degradation, especially in LDL and HDL.

### Glycation and oxidation of LDL in RA patients

LDL from both female and male RA groups showed 17% and 44% higher levels of glycated end products, as shown in Fig 1B. Electrophoresis (SDS-PAGE) revealed that both RA groups showed severe fragmentation of apo-B. Especially, the RAF group showed apo-B_48_ production, as shown in Fig 1C.

**Fig 2:**
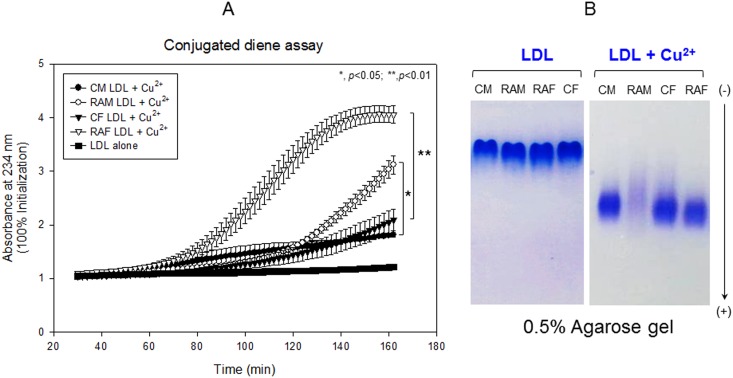
Comparison of LDL oxidation susceptibility by determination of conjugated dienes (A) and Comparison of electromobility of LDL between with or without cupric ion in 0.5% agarose gel (B). CM, control male; RAM, rheumatoid arthritis male; CF, control female; RAF, rheumatoid arthritis female.

Measurement of cupric ion-mediated LDL oxidation revealed that LDL from RAF underwent the most severe oxidation, as shown in Fig 2. After 160 min of incubation, RAM and RAF groups showed 1.7- and 1.9-fold increased conjugated diene levels (Fig 2A). Agarose gel electrophoresis revealed that the RAM group showed almost complete disappearance of the LDL band, whereas LDL from the RAF group showed weaker band intensity than CF (Fig 2B). These results suggest that LDL from RA group was more oxidized and more susceptible to the cupric ion mediated oxidation.

### Glycation of HDL and multimerization of apoA-I

**Fig 3:**
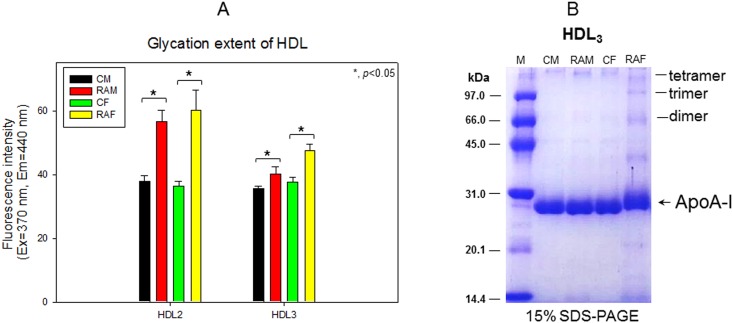
Glycation extent of HDL based on fluorescence determination (A) and electrophoretic profiles of HDL as visualized by Coomassie Brilliant Blue staining (B). CM, control male; RAM, rheumatoid arthritis male; CF, control female; RAF, rheumatoid arthritis female.

All RA groups showed increased glycated end products compared to the control, as shown in Fig 3A. The RAM and RAF groups showed 1.5- and 1.7-fold increased glycated end products. The RAF group showed the most distinct multimerization of apoA-I with a slight upward shift in band position as indicated by the arrow (Fig 3B), suggesting that aggregation occurred by glycation.

### Elevated CETP and reduced PON activity in HDL from RA

**Fig 4:**
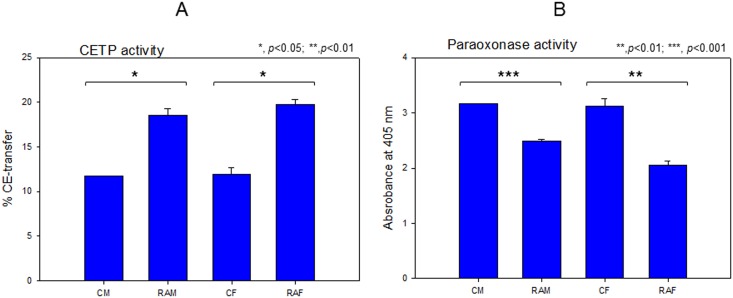
HDL_3_ associated cholesteryl ester transfer protein (CETP) activity (A) and paraoxonase (PON) activity (B). CM, control male; RAM, rheumatoid arthritis male; CF, control female; RAF, rheumatoid arthritis female.

In the HDL_3_ fraction, RAM and RAF groups showed 1.6- and 1.7-fold higher CETP activities than the control group (Fig 4A). The male and female RA groups showed 22% and 34% lower paraoxonase activities than the control, respectively (Fig 4B).

### Uptake of LDL into macrophages

**Fig 5:**
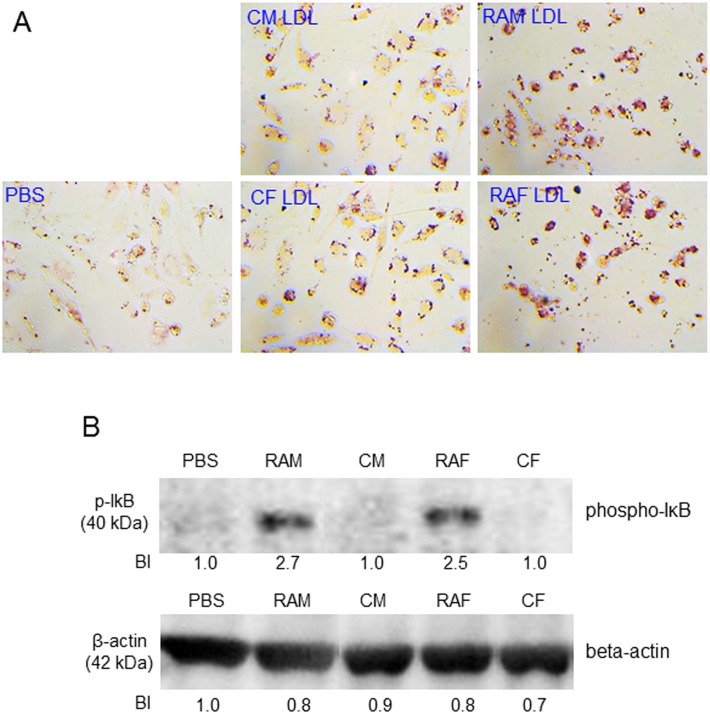
Uptake of LDL from each group into macrophages was visualized by Oil-red O staining to detect fatty acid and triglyceride (A). Immunodetection of phospho-IκB in cell lysate of the macrophages. Lower numbers indicate band intensity from Chemi-Doc analysis (B). Beta actin was immunodetected as a internal standard. CM, control male; RAM, rheumatoid arthritis male; CF, control female; RAF, rheumatoid arthritis female.

During incubation for 48 hours in the absence of cupric ion, LDL from RA patients was more easily phagocytosed by macrophages, as shown in Fig 5A. Calculation of the oil red O-stained area revealed that the RAM and RAF groups showed 2.7- and 3.4-fold higher fatty acid accumulation than the control, respectively. Quantification of malondialdehyde (MDA) as an oxidized species in cell culture media demonstrated that the RAM and RAF groups showed 2.9- and 2.8-fold higher oxidation than the control, indicating that LDL from RA patients induced oxidation of media. After 48 hours, cell number was reduced in the RA patient group, suggesting putative cytotoxicity.

Both RAM and RAF group showed 2.7 and 2.5-fold, respectively, higher phospho-IκB level than each control group, based on densitometric band analysis using Chemi-Doc, while all group showed similar level of beta-actin (Fig 5B). These results suggest that LDL from RA group had more atherogenic and more inflammatory properties.

**Fig 6:**
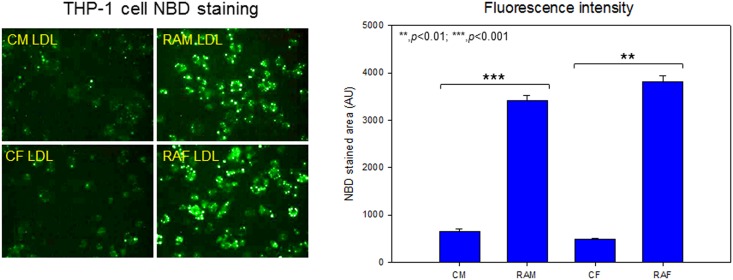
Uptake of LDL from each group into macrophages was visualized by fluorospectroscopy to detect NBD-cholesterol. CM, control male; RAM, rheumatoid arthritis male; CF, control female; RAF, rheumatoid arthritis female.

As shown in Fig 6, phagocytosis of NBD-cholesterol-labeled LDL by macrophages was easily detected to compare extent of atherogenic ability. LDL from the RAM and RAF groups showed 5.3- and 7.9-fold, respectively, higher uptake than the control. These results suggest that LDL from RA patients was more oxidized and easily taken up into macrophages, resulting in foam cell production and cell death, especially in female patients.

### Particle size and number of HDL

**Fig 7:**
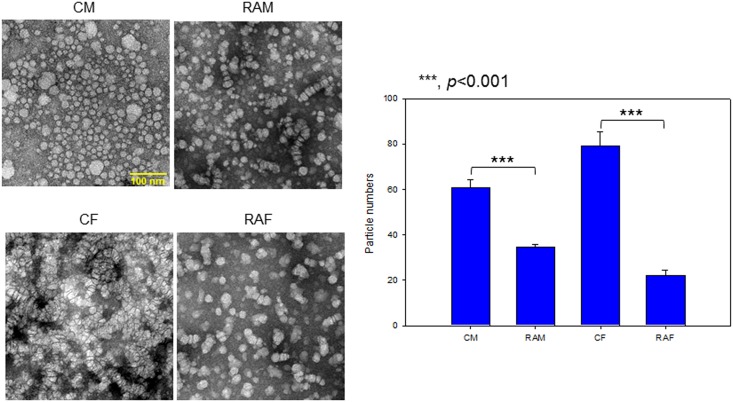
Representative photo of negatively-stained HDL_2_ from RA patients and controls (electron microscopy). All micrographs are shown at a magnification of 40,000×. The scale bar corresponds to 100 nm. Graphs show measured particle size and number from designated area. CM, control male; RAM, rheumatoid arthritis male; CF, control female; RAF, rheumatoid arthritis female.

Electron microscopy, as shown in Fig 7, revealed that HDL_2_ (0.3 mg/mL of protein) particle size was similar between the RA and control groups. However, particle number of RA was remarkably smaller than that of the control. HDL_2_ from RAM showed 23% less particle number than the control. The RAF group also showed 72% reduction in particle number compared to the CF group, although particle size was similar between the groups at around 334–372 nm^2^.

## Discussion

It is well known that the leading cause of death for RA patients is cardiovascular disease (CVD) [32]. Although RA patients frequently are at higher risk of myocardial infarction, stroke, and carotid plaque, they have similar lipid profiles, BMIs, and systolic blood pressures as the control [33]. This result suggests different risk factors such as inflammation, endothelial dysfunction, neutrophilia, and monocytosis. Although plasma lipoproteins are important for maintenance of endothelial cell functions and anti-inflammation, specific biomarkers of plasma lipid level to diagnose RA development and progression have not been reported. This study was designed to investigate unique properties of lipoprotein parameters between male and female RA patients to develop putative biomarkers for RA and CVD risk.

Both RA groups showed significantly lower HDL-C and % HDL-C in TC along with normal levels of TC, LDL-C, and glucose (Table 1).

The RA group showed significant elevation of plasma TG/HDL-C, uric acid, CETP mass, and CETP activity. Elevation of TG in female patients is a unique feature of this study. Female patients showed 2-fold greater TG than the control, whereas male patients showed a similar level as the control (Table 1). Many previous reports showed controversial data about the TG level. A cohort study on the US health plan (n = 30,831 RA patients) showed that RA patients had relatively higher serum TG levels than healthy populations, around 113–156 mg/dL [34]. Another study with Korean patients showed that serum TG level was significantly higher in the Korean RA patient group than the age- and gender-matched control, whereas HDL-C levels were reduced [35]. Similar to the current study, Korean RA patients showed higher serum TG levels, which might be associated with the carbohydrate-enriched dietary pattern of Koreans. At the time of blood donation, around 50% of patients were taking corticosteroid (Predinsolone) at 5.0±3.1 and 2.2±2.7 mg/day for males (n = 4 in 8) and females (n = 13 in 25) patients, respectively. However, many reports have shown no change in lipid and lipoprotein metabolism except a slight increase in HDL-C [36].

Recently, MTX treatment for 6 months was shown to be associated with slight increases in serum HDL along with facilitation of cholesterol efflux [37]. However, a 2-year randomized clinical trial on MTX monotherapy, MTX+ETA combination therapy, and Triple Therapy did not show alteration of HDL-C and TC/HDL-C [38]. Initially, all patients showed impaired HDL function with a high DAS score around 5.5. Similarly, our current RA patients showed low levels of HDL-C and dysfunctional HDL regardless of MTX alone or MTX-based combination therapy. In addition, there was no difference in the consumption of nonsteroidal anti-inflammatory drugs between active and inactive RA patients [39] in change of lipid level.

The hyperuricemia result makes a good agreement with previous report, the Third National Health and Nutrition Examination Survey (NHANES) [40], women self-reporting RA had significantly higher plasma uric acid levels compared to women not self-reporting RA.

Especially, the female RA group showed 2-fold higher plasma IL-6, TG, AST, and ALT levels, whereas the male RA group did not. In the LDL fraction, the female group showed more severe glycation (Fig 1B) and fragmentation of apo-B (Fig 1C). LDL from the RA group showed more cupric ion-mediated oxidation (Fig 2). In HDL_2_ and HDL_3_, the RAF group showed more severe glycation (Fig 3A) and multimerization of apoA-I (Fig 3B). CETP activity was more enhanced in the RA group in both plasma (Table 1) and HDL_3_ (Fig 4A), although the female group showed slightly higher CETP activity than the male group. Phagocytosis of LDL was remarkably accelerated in the RA group (Fig 5), especially in females up to 7.9-fold compared to the control (Fig 6). The RA group showed decreased particle size and number of HDL (Fig 7).

In Fig 2A, RAF-LDL showed the highest production of conjugated diene (A_234_), whereas RAM-LDL, which showed the second highest A_234_ production, almost disappeared in agarose gel electrophoresis. Although it is hard to explain the discrepancy, there was a difference between speed of amino acid degradation and conjugated diene production. There is a possibility that RAM-LDL was more associated with proteolytic enzymes such as MMP-1 and MMP-3, as explained in a previous report [41]

Although it remains unclear why females showed more severely impaired lipoprotein properties than males, RAF showed more atherogenic LDL and dysfunctional HDL. Indeed, it has been known that female:male ratio of RA patients was 3:1 and the most prevalent age was fifties, suggesting that loss of estrogen via menopause might be correlated with production of proinflammatory lipoproteins. Many previous reports suggested the possibility of functional impairment of lipoproteins, such as pro-inflammatory HDL [42]. However, there has been no report comparing functional and structural correlations between patients and controls. In the current study, we compared functional and structural characteristics of lipoproteins from RA patient to identify unique biomarkers. Increased plasma TG and reduced HDL-C levels are well characterized in patients with chronic or acute inflammatory disease, such as end stage renal disease [43] and hemorrhagic fever renal syndrome [44], respectively.

Increased plasma TG and enriched TG lipoproteins (Table 1) are known risk factor of cardiovascular disease and hypertriglyceridemia, which are well associated with inflammatory progression. A reduced HDL-C level via higher CETP activity is the most common lipid abnormality observed in families with premature CHD [45]. TG-rich lipoproteins are pro-atherogenic [46] and an important and independent predictor of CVD and stroke risk in the Asia-Pacific region [47]. Therefore, it is plausible that elevation of TG and CETP in RA patients contributes to increased risk of CVD. CETP activity is a major risk factor of CVD via elevation of TG and reduced HDL-C [48]. In diabetic patients, elevated CETP activity is correlated with large VLDL particles enriched with TG [49].

Although RA is associated with premature cardiovascular mortality, however, in cannot be explained alone by traditional cardiovascular biomarker, such as dyslipidemia and hypertension. Glycation of hemoglobin is a well-known biomarker of diabetic complications, and pentosidine in IgG is associated with RA. In addition, diabetic patients with coronary syndrome showed higher glycation of apoA-I, and reduction of PON activity in HDL [50]. Modification of apoA-I is an important feature of dysfunctional HDL, which is frequently detected in CVD and diabetes. In this report, RA patients showed multimerization of apoA-I in HDL and hyperuricemia. It has been reported that hyperuricemia is independently associated with CVD risk in RA patients from a cross-sectional study [51]. Taken together, hyperuricemia is associated with generation of dysfunctional HDL in RA, which is linked to high risk of CVD. Glycation of HDL is associated with aortic stiffness and endothelial dysfunction because glycated HDL could not prevent phagocytosis of LDL to result foam cell formation [52]. Glycation of lipoproteins is associated with progression of atherosclerosis via reduction of nitric oxide and endothelial cell death.

Very similarly and recently, Botta et al. reported interesting data that active RA patients also showed characteristic features of dysfunctional HDL [53], including high CETP activity, low apoA-I level, and low PON activity. Moreover, active RA patients showed higher arterial stiffness than inactive RA and control subjects.

This report provides new information about emerging risk factors of CVD in RA patients at the lipoprotein level. Increased TG/HDL-C levels and CETP activity were associated with increased uric acid and dysfunctional HDL. These dyslipidemic markers were correlated with impairment of lipoproteins and apolipoproteins (Figs 1–3), resulting in pro-inflammatory functions of LDL in macrophages (Figs 5 and 6). Reduced antioxidant activity in HDL was correlated with more oxidized LDL (Fig 2) and phagocytosis (Figs 5 and 6) in the RA group.

This study is limited in detecting subtle differences in lipoproteins due to the relatively small number of RAM patients. In addition, the serum HbA1c level was not evaluated in this study. Therefore, it was hard to correlate the extent of HDL glycation to predict diabetic conditions of RA patients. For a future study, analysis of arterial stiffness, such as pulse wave velocity measurement from carotid to femoral arteries, should be evaluated to investigate correlations of vascular functions and HDL characteristics between patients and controls.

In conclusion, In addition to traditional biomarkers, the current cross-sectional study details new biochemical features of lipoproteins from RA patients; increased CETP mass and activity, elevated TG content and glycation extent in blood and lipoproteins, lowered antioxidant activity, and multimerization of apoA-I can be useful biomarkers of RA.

## Supporting Information

S1 Table Lipid and protein composition in lipoproteins of rheumatoid arthritis (RA). (DOCX)
