# Supplementary material for: Patients with Rheumatoid Arthritis Show Altered Lipoprotein Profiles with Dysfunctional High-Density Lipoproteins that Can Exacerbate Inflammatory and Atherogenic Process
Source: PLoS One. 2016 Oct 13;11(10):e0164564. doi: 10.1371/journal.pone.0164564 (PMC5063466; doi:10.1371/journal.pone.0164564)
Supplement: S1 Table — (DOCX) [file pone.0164564.s001.docx]

Supplemental Table. Lipid and protein composition in lipoproteins of rheumatoid arthritis (RA)

|  | CM  (n=5, Age=69±4) | | | RAM  (n=5, Age=69±4) | | | CF  (n=3, Age=43±11) | | | RAF  (n=15, Age=53±7.0) | | |
| --- | --- | --- | --- | --- | --- | --- | --- | --- | --- | --- | --- | --- |
|  | TC | TG | TP | TC | TG | TP | TC | TG | TP | TC | TG | TP |
| VLDL  ( % wt ) | 24.1±0.3 | 64.4±0.8 | 11.5±2.3 | 11.2±0.5^*^ | 78.7±0.5^*^ | 10.1±2.1 | 25.0±7.1 | 49.9±20.4 | 25.1±2.3 | 19.9±0.3 | 67.0±0.5 ^*^ | 13.1±2.8 ^*^ |
| LDL  ( % wt ) | 49.5±0.2 | 5.3±0.3 | 45.1±1.1 | 50.8±0.2 | 11.0±0.7^**^ | 38.2±0.7^*^ | 47.6±4.4 | 16.4±3.5 | 36.0±2.3 | 43.2±0.1 | 31.1±0.2^*^ | 25.7±0.8^*^ |
| HDL_2_  ( % wt ) | 30.8±0.5 | 4.5±0.2 | 64.7±3.7 | 34.6±0.7 | 15.8±0.2^**^ | 49.6±1.8^*^ | 32.4±4.2 | 13.3±9.8 | 54.3±3.0 | 34.3±2.0 | 25.2±1.2 ^*^ | 40.5±6.9^*^ |
| HDL_3_  ( % wt ) | 26.5±0.2 | 2.5±0.4 | 71.0±0.4 | 19.5±0.1^*^ | 10.2±0.5^**^ | 70.3±0.8 | 21.3±1.5 | 9.9±3.7 | 68.8±1.2 | 9.3±0.3^**^ | 33.9±0.2^**^ | 56.9±0.4^*^ |

CM, control male; RAM, rheumatoid arthritis male; CF, control female; RAF, rheumatoid arthritis female; TC, total cholesterol; TG, triglyceride; TP, total protein; VLDL, very low-density lipoprotein; LDL, low-density lipoprotein; HDL, high-density lipoprotein.

*, *p*<0.05; **, *p*<0.01
